# Probiotics in Irritable Bowel Syndrome: A Review of Their Therapeutic Role

**DOI:** 10.7759/cureus.24240

**Published:** 2022-04-18

**Authors:** Lakshmi Satish Kumar, Lakshmi Sree Pugalenthi, Mahlika Ahmad, Sanjana Reddy, Zineb Barkhane, Jalal Elmadi

**Affiliations:** 1 Internal Medicine, University of Perpetual Help System DALTA, Manila, PHL; 2 Internal Medicine, Bicol Christian College of Medicine, Legazpi City, PHL; 3 Research, Ziauddin University, Karachi, PAK; 4 Medicine, Bogomolets National Medical University, Kiev, UKR; 5 Research, Faculty of Medicine and Pharmacy of Casablanca, Université Hassan II de Casablanca, Casablanca, MAR; 6 Faculty of Medical Sciences, Universidad Nacional Autónoma de Honduras, Tegucigalpa, HND

**Keywords:** fodmaps, rome iv criteria, gastrointestinal, bloating, flatulence, stool frequency, symptomatic relief, microbiome, irritable bowel syndrome, probiotics

## Abstract

Irritable bowel syndrome (IBS) is a chronic collection of symptoms and lowers the quality of life. The management of such patients has always involved mitigating the symptoms produced by this disorder. This article reviews the role of probiotics in IBS by compiling various studies to deduce the possible symptomatic relief that probiotics may provide to IBS patients. Given the encouraging part of probiotics in abundant other gastrointestinal conditions, this article focuses on understanding the specific functional effects (if any) that are brought about by adding probiotics in patients with different types of IBS such as IBS with predominant constipation, IBS with predominant diarrhea, and even the unclassified type of IBS. The purpose of analyzing the role of probiotics is to study the changes brought about by them at the level of the gut microbiota in patients suffering from IBS, as this may prove to be of prime importance in managing such conditions with time. This article has also furnished an overview of the pathogenesis, diagnostic criteria, treatment modalities, sources of probiotics, and their therapeutic significance in IBS patients.

## Introduction and background

Irritable bowel syndrome (IBS) is a chronic disorder that results in a spectrum of gastrointestinal (GI) symptomatology, and this usually remains a diagnosis of exclusion [1,2]. Several attempts have been made to understand the close association between the increased longevity of individuals and their consumption of fermented dairy products. History claims that the effect of putrefaction (that led to diseases, again) by the gastrointestinal tract could be mitigated by lactobacilli [3-5]. Several studies claim that IBS can be found in 7%-15% of the general population [6,7]. It is found that the prevalence of IBS is the highest in South America (accounting for 21%) [8,9]. Among the geographic regions most studied, South Asia has the lowest prevalence, accounting for 7% of the cases [10-14]. Studies have shown a slight female predominance in the case of IBS compared to males [10].

Probiotics are living microorganisms that are nonpathogenic. They are known to produce several beneficial effects, such as altering the host's immune response in the gastrointestinal tract and lowering the growth of pathogenic organisms by enhancing the microbial balance. These can be consumed in the form of food and even dietary supplements. Several strains are found to be used as probiotics and these include *Lactobacillus*, *Bifidobacterium*, and even *Saccharomyces*. However, the exact count of various species to be used to bring about specific therapeutic gain is not known yet [15].

IBS is a term used to describe a collection of symptoms that results from several etiologies such as increased risk of infections, malabsorption from infectious diseases, small intestinal bacterial overgrowth (SIBO), antibiotic usage, changes in the gut microbiome, and exaggerated motor response leading to diarrhea- or constipation-like symptoms. Several factors leading to psychological stress, such as increased depression, anxiety-related conditions, can also contribute to IBS [16]. The various associations with IBS include psychological stress, smoking, frequent alcohol consumption, and younger age [14]. Evidence suggests that decreasing age also means a decreasing prevalence of IBS [11]. The Rome IV criteria classify patients based on symptoms as IBS with diarrhea (IBS-D), IBS with constipation (IBS-C), and IBS with mixed bowel patterns (IBS-M) [12]. IBS results from pathophysiology that is broad and not yet entirely understood. The abnormal physiology can result in psychological stress and even motility disorders. The treatment goal for IBS is to make the patients feel symptomatically better by relieving symptoms of cramping, pain, diarrhea, constipation, and bloating, thereby helping them improve their quality of life [11]. Exercise can be encouraged to improve the peristaltic function of the intestine in the case of constipation and the use of laxatives, a high-fiber diet, and pro-kinetic drugs [11,13].

IBS is a collection of symptoms with a poorly understood etiology, thereby leading to lower quality of life. Out of the various available options, the use of probiotics in people suffering from IBS is still not clearly understood [15]. One must aim to understand the efficacy of their usage to improve patients' quality of life, thereby being one of the many solutions that relieve symptoms in patients. This article aims to review, from several studies, the efficacy of probiotics in people suffering from a chronic condition such as IBS and determine if modifying the gut's microbiome in such patients has an added benefit or if it instead further worsens the symptoms mentioned above that are already present in patients.

## Review

Pathogenesis

**Figure 1:**
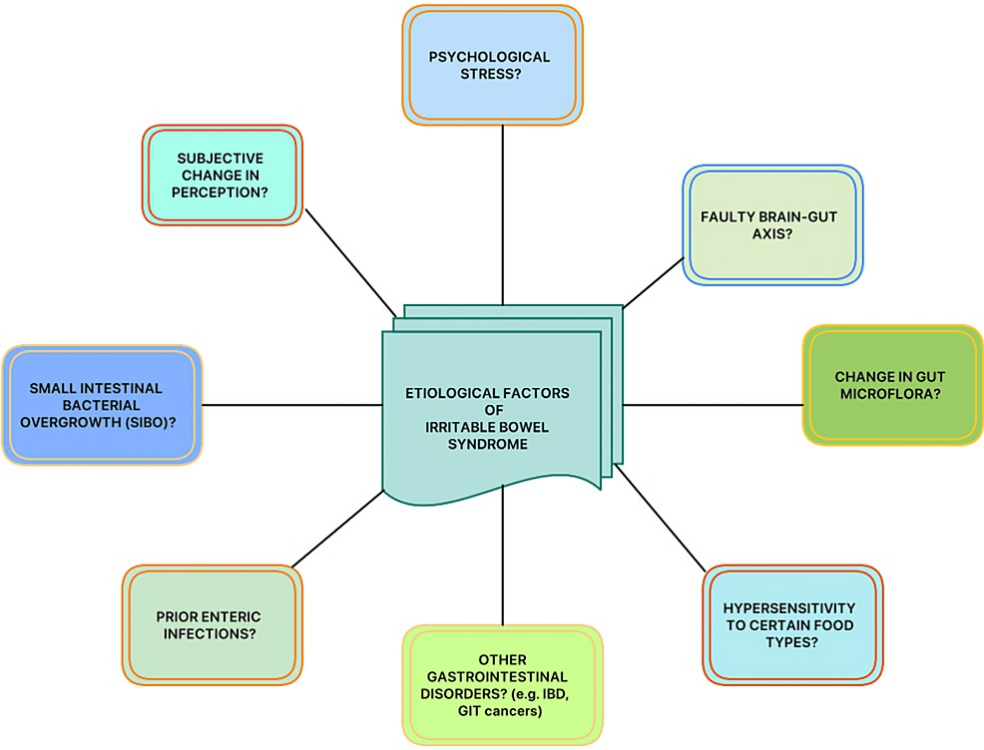
Possible etiological factors of IBS IBS, irritable bowel syndrome; IBD, inflammatory bowel disease; GIT, gastrointestinal tract

Although the pathogenesis and probable etiologies of IBS are still not clear, it is necessary to document the current understanding of IBS and its diagnostic and treatment modalities (Figure 1) [17]. Previous studies have shown that IBS can even be triggered by various factors ranging from psychological stress to enteric infections [18]. Another possible and unique mechanism mentioned is that the gut viscera is thought to be hypersensitive to different types of foods, the types differing from person to person. A large number of existing studies in the broader literature have also examined the female predominance in the case of IBS disorders.

Another relevant mechanism in evidence from the previously published literature is that IBS could also result from an alteration in the microflora of the gut, genetic predisposition to several other gut disorders like inflammatory bowel diseases (IBDs), or even a subjective change in the way people perceive their symptoms and severity, which often leads to complexities in which they are both diagnosed and managed [18].

Some have proposed that it could even involve the brain-gut axis. Several authors have recognized that IBS is a fault in the connection between the brain and the gut [18]. While understanding the cause of IBS is vital (which has been proven to be multifactorial as of today), this article aims to understand the effect that probiotics can have on these patients [19].

With the help of studies conducted to date, it is crucial to scrutinize and further summarize the role of modifying gut flora in IBS patients. Given that hypersensitivity could also be a possible etiology in IBS patients, answers to questions like "Are these patients hypersensitive to added probiotics?", "Do they tolerate them well in case of irritable bowel?" or "Do they worsen in the long run because of a possible dominance of certain bacterial species over the years despite using a broad spectrum of probiotics?" remain unanswered [20]. In the case of IBS patients, the enteric microbiota has become a target for therapeutic purposes, as proposed by several clinical and preclinical studies [21].

Pimentel and Lembo explained the reasons why a change in the gut microflora can result in the common symptoms, such as diarrhea, constipation, a combination of these two, bloating, and abdominal cramps, that patients complain of, all of which reduce their period of productivity during their active years of life as gut microbes exert effects on the host immune system and gut barrier function, as well as the brain-gut axis [22]. A positive hydrogen breath test would increase the bacterial overgrowth and present like a diarrhea-predominant IBS (IBS-D). On the other hand, a positive methane breath test would indicate an excess of methanogenic archaea in the gut presenting as constipation-predominant IBS because the intestinal contractility slows down due to the presence of the methane gas. Cytolethal distending toxin B (CdtB) is a common toxin produced by the microbes responsible for gastroenteritis and those leading to the IBS symptoms. A cross-reaction occurs between the antibodies raised against CdtB and vinculin, a cytoskeletal protein eventually impairing the function of gut motility and thereby further assisting in the overgrowth of bacteria [22].

Stasi et al. even proposed that the brain-gut axis can be affected by several psychological disorders [23]. These modify the motility of the digestive tract, various inflammatory pathways, and visceral sensitivity. This axis can be affected due to the release of the corticotropin-releasing hormone, which influences the mood in several autonomic and neuroendocrine ways. The serological adrenocorticotropic hormone and cortisol increase in cases of stress in IBS patients due to a rise in the pro-inflammatory cytokines [24]. Some studies have even attributed gastrointestinal motility to serotonin, reporting that its levels are increased in those IBS patients suffering from diarrhea. In contrast, those with constipation have a reduced concentration of plasma serotonin. Pharmacotherapy targeting these serotonin receptors might be of therapeutic importance [25,26]. Some studies also mention that abdominal pain can be linked to the hypersensitivity of the viscera [27].

Diagnosis of IBS

There are four major bowel patterns observed in IBS that are, namely, IBS-D (predominant diarrhea), IBS-C (predominant constipation), IBS-M (mixed diarrhea and constipation), and IBS-U (the unclassified type, symptoms of which cannot be categorized into any one of the three subtypes that have been mentioned) [28]. Although the Rome IV criteria have been formulated to diagnose IBS, it is crucial for physicians to clinically analyze the findings and exclude all possible causes before confirming them (Table 1) [29].

**Table 1:** Rome IV criteria for diagnosing IBS The patient should be meeting the criteria for the last three months, with the symptoms beginning at least six months before diagnosis [29]. IBS, irritable bowel syndrome

Abdominal pain that is recurrent at least once a day or week in the last three months, along with a minimum of two of the following criteria:
Defecation-related conditions
Associated with a change in stool frequency
Associated with a change in form (appearance) of stool

There is no individual-specific test to confirm IBS, and it is a combined approach involving factors mentioned in the Rome IV criteria (Table 1). These symptoms may seem atypical and may not be due to any other disorder [17]. Exacerbating factors like stress, other psychological disorders, and family history of other gastrointestinal diseases should be considered and this requires a physician's high index of suspicion after ruling out all other possibilities [17].

Treatment options for IBS

Given that IBS is usually a chronic condition, physicians must primarily focus on more sustainable therapeutic options, pharmacological or non-pharmacological. Currently, there are several non-pharmacological treatment options for IBS patients, the details of which will not be dealt with in this review. The benefits of lifestyle modifications, dietary changes, and probiotics must be analyzed with the help of evidence-based medicine so that clinicians navigate towards a holistic approach to treating patients. Given the benefits of probiotics for general gut health and improving the microflora, it is principal to scrutinize their possible role and side effects in IBS patients who are prone to not tolerating even small changes made to the enteric environment and to test the efficacy of probiotics concerning the common complaints (symptoms) mentioned by IBS patients as this condition significantly leads to a reduced quality of life [30].

A wide range of treatment options has been described in several studies. Dietary modifications for IBS patients include elimination diets (by eliminating foods that exacerbate symptoms), increased dietary fiber, a gluten-free diet, and exercise [31]. A diet with low fermentable oligo-di-mono-saccharides and polyols (FODMAPs) has been recommended to improve the global symptoms associated with IBS-D, such as pain and bloating [32,33]. A low FODMAP diet has also been recommended for patients with IBS as a first-line treatment in primary care [34]. The efficacy of probiotics in IBS has also been tested, and symptoms of bloating, abdominal pain, and flatulence showed significant improvements [35,36].

Probiotics are safe and effective in IBS patients, especially those used for a shorter duration such as for less than eight weeks; a higher dosage of a single probiotic strain seem to show greater benefits. The adverse events associated with probiotics are found to be safer in comparison to many other treatment options available today [37]. Probiotics have been shown to improve overall stool frequency, gut transit time, and stool consistency [38]. *Bacillus coagulans *strain LBSC (DSM17654) has been shown to be efficacious in alleviating IBS symptoms such as bloating, abdominal pain, constipation, diarrhea, nausea, vomiting, and stomach rumbling. It was found to be safe for human consumption and it helped improve the quality of life in IBS patients [39]. Thus, probiotics have an overall positive impact on IBS patients by improving their quality of life [40].

Medications to relieve the pain in IBS patients have been tested, but they come with noted side effects, e.g., antispasmodics (which caused more side effects such as blurred vision, dry mouth, constipation, and dizziness), peppermint oil (leading to symptoms of xerostomia, gastroesophageal reflux, and belching), antidepressants (with side effects like dry mouth and drowsiness), and loperamide (that reduced stool frequency, duration and intensity of pain yet an increased intensity of abdominal pain at night). Camilleri's observations concerning the efficacy of the drugs mentioned above did not show any significant improvement in symptoms [31]. However, some off-label drugs were tried by some studies, e.g., histamine H1 receptor agonist (ebastine) reduced overall IBS symptoms, abdominal pain, and visceral hypersensitivity [41]. GABAergic (gamma-aminobutyric acid) agents showed lower pain scores [42].

Medications for diarrhea include using 5-hydroxytryptamine or serotonin type 3 (5-HT3) receptor antagonists and have shown a significant effect on bloating, frequency, urgency, and stool consistency but no effect on pain sensation [43]. Bile acid sequestrants have also been tried for the same by using colestipol as an open-label treatment, and it showed an improvement in the severity scores of IBS patients and stool frequency [44]. Antibiotics like rifaximin showed an overall improvement in global symptoms and bloating but did not affect the frequency of bowel movements, urgency, or stool consistency [45].

A trial of lubiprostone (a chloride channel-related intestinal secretagogue) proved to improve the abdominal pain in IBS patients, although a common side effect of nausea was observed in them [46]. A 5-hydroxytryptamine or serotonin type 4 (5-HT4) receptor agonist like tegaserod has been shown to be effective in IBS-C patients and has improved productivity in work and quality of life [47].

The above-mentioned treatment modalities aid in appreciating the superiority of probiotics when their adverse events are compared to pharmacotherapeutic agents. Hence, we as researchers must continue analyzing the untapped benefits of probiotics in such chronic conditions to make more appropriate choices that are strain-specific to symptoms in IBS patients.

Sources of probiotics

The familiar sources of probiotics include kefir, yogurt, and some other fermented foods. All of these contain different microorganisms that potentially offer better gut health. The most commonly used organisms in these sources include *Streptococcus thermophilus*,* Lactobacillus *strains*, Lactobacillus delbrueckii *subsp.* bulgaricus*,and *Bifidobacterium* strains. Studies have proven that these enhance intestinal health and overall anti-inflammatory and immune responses [48].

Other than the usual *Lactobacillus* and* Bifidobacterium *species, some commonly used microorganisms in probiotic preparations include the *Enterococcus* and *Streptococcus* species. Different formulations of probiotic products are available, ranging from *Bacillus *species to yeasts (*Saccharomyces cerevisiae *and *S.* *boulardii*) and even *Aspergillus oryzae*, which is a filamentous fungus, all of which can be made available in the form of capsules, powders, pastes, tablets, or sprays depending on the feasibility. The use of probiotics is a more natural approach and has fewer side effects when compared to pharmacotherapeutic drugs [49].

A rising interest in tapping the benefits of probiotics is seen because of understanding what harmful effects can be brought about by the increased use of antibiotics that destroy the gut microbiota [50].

Therapeutic significance and prognostic value

Probiotics have long been indicated in several gastrointestinal conditions ranging from traveler's diarrhea to rotavirus enteritis and even in IBDs, colon cancer, acute pancreatitis, HIV-associated diarrhea, and IBS. Studies have shown that probiotics in the colonic mucosa help in producing essential nutrients, toxin elimination, improve intestinal immunity, prevent microbial translocation, and assist in the recovery of a disturbed gut mucosal barrier [51]. Many of the established effects of probiotics include their benefits in inflammatory diseases concerning the gastrointestinal tract, reduced unspecific complaints by healthy people, and improved stool consistency. Thus, when probiotics are consumed in sufficient amounts, they promote positive effects in the gut [52].

Dimidi et al. observed a decrease in *Lactobacilli* and *Bifidobacterium* and higher breath methane in adults complaining of functional constipation. Certain strains of probiotics can modify processes like secretion and motility of the gut and thereby alter the environment of the lumen [53]. The microbiota composition varies depending on the intestine's environmental conditions, an individual's diet, probiotic bacteria that are ingested, one's exposition to it, and how the host system permits the growth of new strains over a period dynamic [54,55].

A study conducted by Nobaek et al. observed the role of* Lactobacillus plantarum* in reducing the formation of gas in 60 IBS patients for four weeks. The two groups involved one receiving a plain rosehip drink and 400 ml of rosehip drink containing 5 × 10^7^ CFU/ml of *L. plantarum* and 0.009 g/ml oat flour per day. A survey following the randomized control trial (RCT) revealed that it significantly reduced the complaint of flatulence in the test group and reduced abdominal pain in both groups. A better overall GI function had been reported at the 12-month follow-up by patients of the test group. However, there was no evidence that the *L. plantatrum* improved the bloating sensation in both groups [56].

To test the efficacy of probiotics that are commercially made available like VSL#3, a combination probiotic tablet consisting of bacterial strains of eight different types, namely, three strains of *Bifidobacterium *(*B. breve, B. longum, B. infantis*), four strains of* Lactobacillus* (*L. acidophilus, L. plantarum, L. casei, and L. delbrueckii *subsp. *bulgaricus*), and one strain of *Streptococcus *(*S. salivarius *subsp. *thermophilus*) on the various symptoms and colonic transit in patients with IBS. Kim et al. conducted a randomized, double-blind study in patients with IBS and bloating for a treatment duration of four and eight weeks. The study results mentioned that VSL#3 reduced flatulence and retarded the colonic transit without changing the function of the bowel in the same patients. For aspects of bloating, abdominal pain, and stool-related symptoms, the study did not observe satisfactory relief by the responders [57].

Dolin performed a randomized, double-blind, placebo-controlled clinical trial to test the role of probiotics in IBS-D and evaluate the effects of a probiotic that contained *Bacillus coagulans *GBI-30, 6086 in the same group of 52 patients over eight weeks during which they received either placebo or* Bacillus coagulans *GBI-30, 6086 each day. The study yielded a good outcome by significantly reducing the average number of bowel movements per day in comparison to the placebo group (P=0.042) [58].

Guglielmetti et al. conducted an RCT to analyze the therapeutic role of probiotics in IBS patients. They specifically tested the efficacy of *Bifidobacterium bifidum* MIMBb75 in 122 patients who were given either placebo (N=62) or MIMBb75 (N=60) once every day for four weeks. To assess the severity of the symptoms, they used a 7-point Likert scale, and the outcome involved a reduction in the global assessment of IBS symptoms. The IBS symptoms of pain (discomfort), distention (bloating), digestive disorder, and urgency significantly improved. Forty-seven percent of the patients given* Bifidobacteria *reported adequate relief. In comparison, only 11% of patients in the placebo group reported so (P<0.0001) [59].

Mezzasalma et al. carried out a randomized, double-double-blind parallel-group trial on 150 subjects with IBS-C to test the efficacy of two multispecies probiotic formulates. The subjects were divided into three groups (the third group being the placebo). For 60 days, probiotic formulations were administered to each group daily. This study concluded that the IBS-C patients responded well to each symptom, and a similar response was noticed even during the follow-up period when compared to the placebo group. It concluded that the supplementation of multispecies probiotics is effective in the IBS-C subjects [60].

The efficacy of *Clostridium butyricum* in treating IBS-D was tested by Sun et al. in 2018. It was a randomized, double-blind, prospective,multicentric, placebo-controlled trial and involved a trial of *C. butyricum* or placebo in a group of 200 patients for four weeks. The study revealed that the overall symptoms, stool frequency, and quality of life improved in IBS-D patients when treated with probiotics containing this particular species of the *Clostridium *group [61].

Yoon et al. conducted a study based on the Rome III criteria to analyze the efficacy of multispecies probiotics in patients suffering from IBS. It involved 49 IBS patients and was a randomized, double-blind, placebo-controlled trial. Patients were divided into two groups randomly and were either assigned placebo or multispecies probiotics twice a day for four weeks. The probiotics group was relieved substantially of the symptoms such as abdominal pain/discomfort, bloating, consistency, and stool frequency at the end of the four weeks. The multispecies probiotics were a mixture of *B. longum*, *B. bifidum*,* B. lactis, L. acidophilus, L. rhamnosus*, and *S. thermophilus *[62].

Saggioro et al. performed a clinical trial in 2004 that mentioned a possible role of the short short-term course of *L. plantarum* LP 01 and *B. breve* BR03 or *L. plantarum *LP 01 and LA 02 in treating the pain in different abdominal locations and severity associated with the various IBS symptoms. The trial based on the Rome II criteria was carried out for four weeks on 70 patients (31 males and 39 females) with a mean age of 40 years [63].

The effects of *Escherichia coli *strain* *Nissle 1917 (EcN) in IBS patients were studied by Kruis et al. through a prospective and double-blind study, using the Rome II criteria on 120 subjects for 12 weeks. The outcome was measured using the Integrative Medicine Patient Satisfaction Scale. They were randomized to either receive EcN (n=60) or a placebo (n=60). Although the responder rate was greater in the EcN group, there were significant differences only after weeks 10 and 11, with the best responses from that patient with the usage of antibiotics and gastroenteritis before the onset of IBS (prior alteration in the enteric microflora) [64].

The role of Medivac DS (consisting of *Bacillus subtilis, Streptococcus faecium*) was analyzed by Kim et al. on 40 patients with IBS. It was a double-blind, prospective study in which 20 subjects received a placebo while 20 of them received the Medivac DS probiotic for a treatment period of four weeks. The frequency and severity of abdominal pain decreased significantly in the Medivac DS group (Tables 2, 3). Thus, it proved its role in treating abdominal pain in IBS as it did not lead to any adverse events [65].

**Table 2:** Summary of included studies establishing the effect of probiotics in IBS patients GI, gastrointestinal; IBS, irritable bowel syndrome; IBS-C, IBS with predominant constipation; IBS-D, IBS with predominant diarrhea

Author	Study design	Study population	Salient remarks
Nobaek et al., 2000 [56]	Randomized control trial	60 IBS patients with a regular colonoscopy/barium enema	Flatulence reduced significantly in the test group. Abdominal pain reduced in both groups. Better overall GI function was observed during the follow-up.
Saggioro, 2004 [63]	Clinical trial	70 IBS patients (31 males and 39 females) with a mean age of 40 years, including patients ranging between 26 and 64 years of age	Efficacious in reducing the severity of abdominal pain after a short course of probiotic therapy, responses that were checked on days 14 and 28 proved beneficial in IBS patients.
Kim et al., 2005 [57]	Randomized control, double-blind	IBS patients	Reduction in flatulence and colonic transit time without altering bowel function.
Kim et al., 2006 [65]	Double-blind, prospective study	40 IBS patients	Marked reduction in the frequency and severity of abdominal pain in the subjects (P=0.044, P=0.038) but not in the placebo group.
Dolin et al., 2009 [58]	Randomized, double-blind	52 IBS-D patients	Significantly reduced the average number of bowel movements (P=0.042).
Guglielmetti et al., 2011 [59]	Randomized control trial	122 IBS patients	Improvement in pain, distention, urgency, and digestive disorder (P<0.0001).
Kruis et al., 2012 [64]	Double-blind, prospective study	120 IBS patients	Significant improvement noted to be Δ20.0% points (95% CI 2.6, 37.4), P=0.01, and Δ18.3% points (95% CI 1.0, 35.7), P=0.02, after 10 and 11 weeks, respectively. Best responses were from those with prior alteration in enteric microflora (Δ45.7% points, P=0.029) and also showed greatest change in all groups.
Yoon et al., 2014 [62]	Randomized, double-blind trial	49 IBS patients (probiotics: 25, placebo: 24)	Symptoms such as abdominal pain/discomfort, bloating, stool frequency, and consistency were relieved in the probiotics group compared to placebo (68% vs. 37.5%; P<0.05).
Mezzasalma et al. 2016, [60]	Randomized, double-blind, three-arm parallel-group trial	150 IBS-C patients	Patients responded well to each symptom. Effectiveness proved well.
Sun et al., 2018 [61]	Randomized, double-blind, prospective study	200 IBS-D patients	Overall symptoms, stool frequency, and quality of life improved.

**Table 3:** Summary of included studies indicating prognostic significance IBS, irritable bowel syndrome; IBS-C, IBS with predominant constipation; IBS-D, IBS with predominant diarrhea

Author	Prognostic outcomes
Nobaek et al., 2000 [56]	Reduced flatulence, abdominal pain, and bloating. Also, an overall improvement in gastrointestinal functions was observed.
Saggioro, 2004 [63]	Reduction in general pain at different abdominal locations and severity of various IBS symptoms.
Kim et al., 2005 [57]	Reduced flatulence and colonic transit time without altering bowel function.
Kim et al., 2006 [65]	The frequency and severity of abdominal pain reduced significantly.
Dolin et al., 2009 [58]	Reduced average number of bowel movements per day.
Guglielmetti et al., 2011 [59]	Reduction in the global assessment of IBS symptoms like pain, bloating, digestive disorder, urgency; adequate relief.
Kruis et al., 2012 [64]	Those with a prior history of gastroenteritis and antibiotics usage showed the best responses. Significant differences were recorded after about 10-11 weeks of probiotic use.
Yoon et al., 2014 [62]	Abdominal pain/discomfort, bloating were relieved substantially; stool frequency and consistency improved.
Mezzasalma et al., 2016 [60]	A better outcome was noticed in IBS-C subjects as it improved each symptom, including the follow-up period.
Sun et al., 2018 [61]	Overall symptoms, stool frequency, and quality of life improved in IBS-D patients.

Hence, the prognostic role of probiotics in IBS has to be widely researched as they may have numerous potential benefits given the chronic course of the disorder and pharmacological management of the same population could lead to numerous side effects in a due period. It is critical to realize the above-mentioned utility benefits of probiotics in IBS and encourage their users to tap the several advantages they holds. Future investigations of probiotics in IBS patients should be aimed at identifying specific strains in relieving their symptoms.

Limitations

Given the pathophysiology of IBS, it is beyond the scope of this article to establish a confirmed etiology of the disorder as of today. Also, we have reviewed studies published over the years on only a few strains used in probiotic mixtures. Thus, the efficacies of the different species or their combinations used cannot be compared. This review has strictly limited its discussion to the usage of probiotics in IBS while not extending it to the role of lifestyle modifications and other supportive therapeutic interventions that complement a sooner recovery in the long course of IBS.

## Conclusions

The primary objective of this review was to understand what role probiotics have (if any) in those suffering from IBS. It has been analyzed that several strains mentioned in the review have their functions of relieving and alleviating flatulence abdominal pain, including its frequency and severity, distention (bloating), digestive disorders, and urgency of the bowel. Probiotics have also proven beneficial in IBS patients by slowing down the transit time of the colon, reducing the average number of bowel movements per day, improving stool consistency, overall symptoms, and above all, the quality of life in these patients. It is noteworthy that adding probiotics to IBS patients' routine brings about symptomatic relief. A variety of strains in the probiotic mixtures have shown their benefits. Therefore, we conclude that probiotics have a beneficial role in chronic disorders like IBS. The future implications include encouraging researchers to be involved in more and more studies that can further help dictate management plans for IBS patients by possibly combining a few of these strains, given their benefits, and analyzing their competitiveness when used together in the enteric environment. Strain-specific probiotic usage targeting symptomatic management in IBS patients would be the next big leap in the field of gastroenterology.
